# (Mis)understanding alcohol use disorder: Making the case for a public health first approach

**DOI:** 10.1016/j.drugalcdep.2023.111019

**Published:** 2023-11-04

**Authors:** James Morris, Cassandra L. Boness, Robyn Burton

**Affiliations:** a London South Bank University, Centre for Addictive Behaviours Research, UK; b University of New Mexico, Center on Alcohol, Substance use, And Addictions, USA; c Institute of Psychiatry, Psychology & Neuroscience, King’s College London, UK

**Keywords:** Alcohol, Alcohol use disorder, Biopsychosocial, Framing, Public health

## Abstract

‘Alcohol use disorder’ (AUD) is used by several contemporary conceptualizations to identify, treat and prevent problems associated with alcohol use. Such conceptualizations encompass diagnostic classifications and broader frameworks for policy and practice. However, current AUD concepts are subject to multiple tensions and limitations in capturing and responding to the complex and heterogeneous nature of alcohol problems. Further, *public* understandings of alcohol problems are heavily divergent from professional AUD concepts and remain embedded within an ‘alcoholism’ master narrative in which disease model stereotypes come with multiple costs for prevention and ‘recovery’. The persistence of a problematic ‘alcoholism’ paradigm reflects the coalescing of multiple forces including the cognitive appeal of reductionism, motives to stigmatize and ‘other’, and an overemphasis on AUD as an individually located biomedical problem. Public misperceptions of AUD as a matter of the individual, the individual’s essence, and misconceived notions of responsibility and control have been bolstered by industry interests and the ascension of neuroscience and genetics, in turn diverting attention from the importance of the environmental and commercial determinants of health and the effectiveness of under-utilized public health policies. We call for multiple stakeholders to support efforts to prioritize a public health first approach to advancing AUD research, policy and treatment in order to make significant advances in AUD prevention and treatment. We offer several recommendations to assist in shifting public understanding and scientific limitations in AUD concepts and responses.

## 1 Introduction

*Alcohol Use Disorder* (AUD) has emerged as a contemporary term which reflects several conceptualizations for problems associated with alcohol use, though lay discourses around alcohol problems are largely discordant with AUD conceptualizations (Morris et al., 2022). For example, the *Diagnostic and Statistical Manual for Mental Disorders, Fifth Edition* (DSM-5) identifies AUD as either mild, moderate, or severe depending on the number of criteria met across four conceptual symptom clusters (impaired control, social impairment, risky use, and pharmacological criteria) (APA, 2013; pp. 483–484) (American Psychiatric Association, 2013). The World Health Organization’s current 11th *International Classification of Diseases* (ICD) differentiates between harmful and dependent alcohol use as different categories of AUD and includes a separate non-AUD category of hazardous use (WHO, 2018). The United Kingdom’s National Institute of Health and Care Excellence (NICE) uses AUD as an umbrella term essentially encompassing all ICD-11 categories including hazardous use (NICE, 2011). Accordingly, NICE’s approach to AUD is broadly aligned with the Alcohol Use Disorder Identification Test’s (AUDIT) (Babor et al., 2001) ordinal categorization (i.e., varying cut scores indicative of increasing risk from lower-risk use, to probable dependence), although NICE also identifies severities of dependence. Thus, AUD as a term or label represents differing conceptualizations attempting to capture alcohol problems.

Varying approaches to operationalizing AUD reflect both the inherent complexity and heterogeneity of AUD, in addition to the social, political, and cultural influences on AUD conceptualizations (Boness et al., 2021, 2022; Morris et al., 2023b; Room, 1985). Indeed, the history of ‘alcohol problem’ conceptualizations demonstrates their ongoing evolution as strongly embedded within their socio-cultural context (Boness et al., 2022; Room, 2001). For example, folk concepts of ‘alcoholism’ are still prevalent in current public discourse, reflecting the dominance of the dispositional disease model through most of the 20th Century and ongoing recognition of Alcoholics Anonymous (AA) worldwide (Miller and Kurtz, 1994; Morris, 2022). However, disease concepts of AUD were, and remain, highly contested in the context of the various alcohol and addiction narratives at play (Heather et al., 2018; Pickard, 2022). For instance, the ‘alcoholism’ model came to prominence via the rapid growth of AA following its inception in 1930’s America, alongside the evolution of a medical model of alcohol problems (Heather and Robertson, 1997; Room, 1984). In part, both perspectives sought to address the shortfalls of the preceding moral model of AUD, albeit from different standpoints. Subsequently, the 1980 s saw the influential *alcohol dependence syndrome* (Edwards and Gross, 1976) and operationalization of non-pathological alcohol ‘abuse’ patterns in the DSM-III and ICD-9, reflecting more empirically derived attempts at AUD conceptualizations (Day and Morris, 2021). However, these developments also marked the emergence of a tension between the ‘two worlds of alcohol problems’ as an unintentional splitting of treatment orientated models from broader population focused public health approaches (Storbjörk and Room, 2008). Meanwhile, public discourses have also been in flux, for example, with more contemporary moral panics around ‘binge drinking’ (Herring et al., 2008), and the recent emergence of modern temperance ‘positive sobriety’ identities aided by the use of digital technologies in everyday life (Thurnell-Read and Monaghan, 2023).

Today, tensions around AUD concepts and public narratives related to alcohol use and problems remain. These include ongoing competing perspectives around the weight of focus given to identifying different etiological perspectives of AUD and corresponding funding decisions (Ochterbeck et al., 2023). For example, biomedical approaches such as neuroscientific ‘brain disease’ models and genetic aetiological approaches to AUD have received significantly greater funding allocations in recent decades (Midanik, 2004; Room, 2021). Critics argue a biomedical focus drives individualistic and potentially pathologizing perceptions of AUD, harming recovery outcomes and upstream prevention efforts without delivering significant clinical advances (Lantz et al., 2023; Morriset al., 2023; Room, 2021). Those endorsing biomedically focused conceptualizations claim the unlocked potential for neuroscientific and genetic advances in the treatment and prevention of AUD, whilst suggesting a *biopsychosocial* model adequately considers sociocultural and psychological factors (e.g., MacKillop et al., 2022). Inevitably, calls for other forms of pluralism, agnosticism, or more dynamic context-dependent models, are also offered as potential responses to the various tensions over what AUD, addiction and other problems of living ‘exist’ as (Heather et al., 2022; Pickard, 2022).

In this paper, we evaluate contemporary approaches to AUD conceptualizations and their implications for research, policy, and practice. We aim to address issues with current AUD paradigms, which we argue are overly focused on biomedical causes and responses and overlook important epidemiological evidence showing a strong association between the overall levels of alcohol consumption in a population and the proportion of heavy drinkers. The biomedical model’s inherent emphasis on the individual has serious consequences for the uptake and implementation of crucial public health responses which aim to shift the entire distribution of drinkers downward (including the heaviest drinkers) by targeting key social and environmental determinants of AUD. We attempt to highlight both the strengths and limitations of current models, evaluate new and other proposed models for AUD, and assess how AUD may be best represented or addressed in different contexts. We conclude with reflections on what the future for AUD conceptualizations may hold. We also propose some recommendations for various partners invested in AUD prevention and treatment to pursue in supporting a shared top-level aim of preventing and reducing harm related to alcohol use and AUD.

## 2. Empirical limitations to ‘biopsychosocial’ AUD concepts

Biomedical and biopsychosocial models of AUD tend to focus heavily on biological (e.g., genetic) factors and conceptualizations (e.g., brain disease model) that cause and maintain AUD. Although some of these models, such as the biopsychosocial model, purport to address psychological and social aspects of AUD, individual biogenetic factors are often prioritized most heavily (e.g., MacKillop et al., 2022; Morris et al., 2023; Room, 2021). As a result, biomedical descriptions of AUD only capture a narrow part of the known heterogeneity in AUD (e.g., compulsion; Heather, 2017), thus neglecting other salient factors that cause and maintain AUD (Boness et al., 2021; Kendler, 2012; Pickard, 2022). Biomedical attributions risk emphasizing AUD as an individually located, necessarily chronic problem, which in turn may promote certain stigma processes (Dar-Nimrod and Heine, 2011; Haslam and Kvaale, 2015; Morris, 2022; Morris et al., 2021; Tucker, 2020; Wiens and Walker, 2015).

By virtue of its approach, the biomedical model response emphasizes individualized clinical interventions and overlooks the strong relationship between the overall level of alcohol consumption in a population and the proportion of heavy drinkers within it (Babor et al., 2022; Caetano and Cunradi, 2002). Across countries and over time, the distribution of alcohol consumption is fairly fixed whereby, at the population level, the majority of people drink low to moderate amounts of alcohol with smaller numbers of people drinking more extreme amounts – often referred to as the *distribution of consumption model* (Brunborg et al., 2017; Caetano and Cunradi, 2002; Kehoe et al., 2012; Raninen and Livingston, 2020; Room and Livingston, 2017; Rossow and Clausen, 2013; Skog, 1985). Further, not all people who drink to more extreme amounts will become dependent and those who do often recover without formal treatment (Klingemann and Klingemann, 2018; Witkiewitz et al., 2020; Witkiewitz and Tucker, 2020). This is in direct conflict with the notion that AUD is a chronic and relapsing brain disease. The close connection between mean population-level consumption, the consumption distribution, and the prevalence of heavy drinking suggests that when mean population-level alcohol consumption changes, so does consumption across *all* drinkers in that population. Skog (1985) described these collective changes in population-level alcohol consumption as drinkers “mov[ing] up and down the scale of consumption” (p. 97) in unison. Through social interaction, the alcohol consumption of an individual directly or indirectly affects the alcohol consumption of others, thereby leading to collective changes across drinkers at all consumption levels.

The distribution of consumption model has important implications for public health strategies since it suggests actions which reduce mean levels of consumption in a population will also reduce the prevalence of heavy drinking (Rose, 2001). The most effective interventions to reduce alcohol harm include increases in the price of alcohol, restrictions on marketing, and reductions in the availability of alcohol, all of which reduce alcohol consumption across all drinkers, including the heaviest (Babor et al., 2022; Burton et al., 2017). These actions also likely reduce the likelihood of transition from lower risk levels of drinking through to AUD. When AUD is primarily viewed through the biomedical lens, these highly effective, population-level interventions are easily overlooked, and may indeed account for their under-utilization as policy levers (Burton et al., 2017; Lee, 2023; Morris et al., 2023; Morris et al., 2023b; NICE, 2010).

Alcohol harm arises from the volume and frequency of alcohol consumption, which interacts with individual and societal-level factors. For example, a single occasion of heavy drinking increases the risk of injury (Taylor et al., 2010), whereas heavy consumption over a longer period increases the risk of AUD or conditions such as alcohol-associated liver disease (Rehm et al., 2021). For some alcohol-related conditions such as cancer, the dose-response relationship is linear, with risk increasing at levels above zero intake, thus the larger number of moderate drinkers account for the majority of alcohol-related cancer cases, and heavy drinkers account for the minority. In contrast, the dose-response relationship for cirrhosis is exponential, meaning the burden of liver disease is concentrated among the comparatively smaller group of the heaviest daily drinkers. By focusing only on the heaviest drinkers, as tends to occur through a biomedical focus, there is a risk that the significant harm experienced by the relatively larger number of less heavy drinkers, is overlooked, as are the population-level policies designed to reduce harm across the spectrum of drinkers.

## 3. Advancing accuracy in AUD diagnosis and etiology

Many individuals have advocated for AUD conceptualizations and corresponding diagnoses to focus on the wider range of etiologic and maintenance factors to extend beyond the biomedical model. This includes psychological, social and environmental factors, and those that characterize AUD as a complex and heterogeneous disorder (Boness et al., 2021; Heather, 2017; Kendler, 2012; Pickard, 2022; Room, 2001; Witkiewitz et al., 2020). Such broad conceptualizations are critical given that “the functional significance of genetic variants and polygenic risk scores for AUDs…is largely unclear” and “neuroimaging research has not yet generated clinically informative indicators for improving diagnoses, prognosis or treatment planning” (MacKillop et al., 2022, p.18), rendering the biomedical model incomplete at best for AUD treatment and prevention. Further, advancing biomedical treatment approaches has a range of ethical implications including the potential to increase health inequities, for instance, because structural drivers of poor health are overlooked when seeing conditions as individually located and produced (Barr and Meyers, 2023; Lantz et al., 2023). Indeed, it is becoming increasingly clear that accuracy and precision in AUD diagnosis and treatment for a given person requires explicit consideration of the various processes (e.g., psychological, social, environmental) that cause and maintain AUD, consistent with a precision medicine approach (Boness et al., 2021; Boness and Witkiewitz, 2022; Kwako et al., 2016; Litten et al., 2015). Thus, improved diagnostic accuracy and precision requires going beyond contested narrow conceptualizations of AUD as a chronic, relapsing brain disease (Heather et al., 2022), particularly given key features of this conceptualization (e. g., loss of control, compulsive use) may only be applicable to certain subgroups of people with AUD (Boness et al., 2021). This must also be accompanied by an understanding of socio-political influences on AUD conceptualizations (Boness et al., 2022) and a greater emphasis on environmental factors and the effectiveness of population-level public health approaches (Lantz et al., 2023; Morris et al., 2023b).

## 4. The persistence of AUD stigma and harmful recovery stereotypes: A consequence of the disease model?

People with AUD are heavily stigmatized by the public (Kilian et al., 2021), serving as a significant but under-addressed barrier to the implementation of evidence-based public health approaches (Morris and Schomerus, 2023). Notably, people perceived to have AUD are negatively stereotyped, particularly by being seen as unpredictable, dangerous and blame-worthy, in turn experiencing multiple forms of discrimination (Crisp et al., 2005; Kilian et al., 2021; Schomerus et al.,2022). Awareness of this public stigma has multiple harmful consequences for people with AUD, particularly via internalized stigma (i.e., self-stigma) often leading to guilt, shame, decreased self-esteem, lower self-efficacy, and poorer recovery outcomes (Morris and Schomerus 2023). One stigma coping strategy is *label avoidance*, whereby people escape stigma by resisting a problematic drinking identity (Carrieri et al., 2022; Glass, 2013). For instance, the term ‘alcoholic’ was associated with higher implicit and explicit stigma ratings amongst a general population sample (Ashford et al., 2018), and lower problem recognition amongst a community sample of people drinking at harmful levels (Morris et al., 2021). Indeed, stigma has been consistently identified as the major barrier to help-seeking, contributing to a significant treatment gap where just 1 in 6 people with alcohol dependence receive treatment (May et al., 2019; Mekonen et al., 2021).

This high degree of stigma has been attributed to the common dichotomization of ‘problem’ versus ‘non-problem’ drinkers in lay understandings (Morris et al., 2022; Schomerus, et al., 2011). This binary conceptualization stems in large part from the cognitively appealing ‘alcoholic’ vs ‘not’ heuristic which evolved with the rising popularity of dispositional alcoholism-orientated models in the 20th Century (Babor, 1996; Heather and Robertson, 1997; Pattison et al., 1977), and notably, within the context of an increasingly biomedical approach to health and health care (Lantz et al., 2023). Further, it has been argued that emphasis on a clear delineation between problem and non-problem drinkers has been purposely driven by alcohol industry interests to stifle effective population policies (e.g., McCambridge et al., 2021). However, it has also been proposed that lower severity AUD groups may also drive the dichotomization of problem drinking via *othering* those perceived as problem drinkers in order to maintain their own drinking as normative (Morris et al., 2021; Schomerus et al., 2011). Indeed, multiple drinking groups have demonstrated the othering of ‘different’ drinking groups, notably by pointing to their failures in maintaining *responsibilities* or *control over alcohol*, in turn diverting scrutiny of their own consumption levels (e.g., Davies et al., 2022; Melia et al., 2021). This appears most evidently in the othering of ‘alcoholics’, drawing on extreme stereotypes of AUD as a severe, uncontrollable and pathological condition (Morris, 2022; Wallhed Finn et al., 2014).

In this way, many people with less severe forms of AUD inadvertently perpetrate alcohol stigma by emphasizing difference and essentialism towards the outgroup of ‘problem drinkers’ (Morris and Schomerus, 2023). Some empirical support for this has been identified whereby alcohol stigma is more pervasive in countries with higher consumption, thus questioning claims that stigma may serve as protective factor (Kummetat et al., 2022). This may in part explain the failure of disease models, most recently in the form of a *brain disease model of addiction*, to reduce public stigma towards those perceived to have AUD (see Morris, 2022 for a review). For instance, despite rising public endorsement of addiction as a disease, high levels of alcohol stigma have remained, and on some measures even increased (Pescosolido et al., 2010; Schomerus et al., 2011; Schomerus et al., 2014a). Thus, whilst a disease model may alleviate some stigma components (e.g., blame) in some contexts (Schomerus et al., 2014b, 2022) and among some groups (e.g., AA members or friends and family members of people with addiction; Pickard, 2022), biomedical attributions towards mental health and addiction have been identified as *mixed blessing* models (Haslam and Kvaale, 2015). A mixed blessing account however rests largely on the basis that disease models reduce blame as per attribution theory (Kelley and Michela, 1980), but such findings are both empirically inconsistent^1^ and questionable as a rationale for maintaining such a model (Morris, 2022; Morris and Schomerus, 2023). Rather, emphasizing psychological and societal facets of AUD (McGinty and Barry, 2020; Rundle et al., 2021), continuum models (Morris et al., 2023b; Peter et al., 2021), context dependent framings (Pennington et al., 2023), or other models of responsibility (Pickard, 2017; Schomerus et al., 2022) are proposed as more promising approaches.

Whilst disease models have failed to reduce public stigma towards AUD, it has been argued that AUD stigma is – at least in part – *produced through* biomedical attributions (Morris, 2022). A core facet of public stigma is the process of separation whereby people with the stigmatized condition are marked as *different* and subject to labelling and discrimination (Link and Phelan, 2001). This categorization of people with AUD as fundamentally different is thus facilitated by a disease model where the existence of the disease represents the person’s fundamental essence (Buchman et al., 2011; Harden, 2022). Such essentialism is thus a fundamentally a stigmatizing process (Dar-Nimrod and Heine, 2011; Link and Phelan, 2001; Stupak, 2021) whereby the ‘alcoholic other’ is marked as different, and deemed of ‘bad character’ (Hamilton et al., 2023). Whilst a distinct *alcoholic identity* is a core component of AA (Glassman, et al., 2022) and offers the potential for resolving self-stigma via in-group identification and solidarity (Cruwys and Gunaseelan, 2016), AA’s effectiveness appears to stem mainly from social network support processes (Kelly et al., 2020). Further, whilst an ‘alcoholic identity’ may be empowering for many AA members, this experience is not universal and others may “exit disappointed” (Glassman, et al., 2022) or conceal this identity to avert public stigma threats (Romo et al., 2016). Importantly, from a population perspective, the majority of people who meet AUD criteria will never contemplate adopting an ‘alcoholic identity’ and its embedded implication of being *fundamentally different* from others (Buchman et al., 2011; Morris, 2022); as AA’s main text states, “the delusion that we are like other people, or presently may be, has to be smashed” (Alcoholics Anonymous, 2001, p.30).^2^

Such essentialism is closely aligned with cognitive biases of reductionism and determinism that also contribute to harmful beliefs about being ‘disordered’ (Harden, 2022). For example, believing oneself to have a genetic predisposition to ‘alcoholism’ can induce a reduced sense of control over one’s drinking (Dar-Nimrod et al., 2013), whilst disease model beliefs are associated with the *abstinence violation effect* (Heather et al., 1982) and an increased likelihood of a return to problematic drinking (commonly – but problematically – termed ‘relapse’; Sliedrecht et al., 2022) (Miller et al., 1996). Genetic attributions have been associated with other costs, for example, believing oneself *not* to have a genetic disposition towards ‘alcoholism’ is associated with greater dismissiveness of AUD associated harms (Ahn and Perricone, 2022). Further studies have highlighted how disease orientated ‘alcoholism’ beliefs are prone to *fixed mindset* costs, thus undermining self-efficacy, stigma and recovery outcomes (Lindgren et al., 2020; Wiens and Walker, 2015). Such findings are consistent with behavioral theories in which beliefs about a problem – particularly it’s perceived severity and controllability – are important drivers in their identification and resolution (e.g., Hagger et al., 2017), including for AUD problem recognition and recovery (Bradshaw et al., 2017; Cooke et al., 2016; Corte, 2007; Lindgren et al., 2016; Morris et al., 2021). Thus, outside of AA, a disease embedded concept of AUD appears to have harmful consequences for public stigma, problem recognition and prognostic optimism. These costs seem to significantly outweigh some ‘blessings’ in some contexts (e.g., attenuating blame), particularly considering the persistence of public stigma towards people with AUD *despite* rising disease model attributions (Morris and Schomerus, 2023).

## 5. Advancing a continuum model of AUD: re-framing the master narrative?

In recent years, renewed calls have been made for advancing a continuum aligned model of alcohol use and problems for furthering public health goals. Specifically, Morris, Boness and Witkiewitz (2023) reviewed the evidence for advancing a continuum model in response to the aforementioned limitations to current AUD concepts. Whilst further research is called for, the authors conclude that seeking a *public* understanding of alcohol use and problems as a broad continuum^3^ should be pursued, albeit that their true nature is so highly heterogeneous that it cannot be accurately understood as existing on a single continuum (rather, AUD compromises multiple etiologic factors and consequences, thus *scientifically* exists across multiple continuums, i.e., as multidimensional) (Boness et al., 2021; Watts et al., 2021). Promoting continuum based understandings amongst the public therefore has significant potential for addressing major barriers to progress in reducing AUD, and can be achieved without undermining treatment agendas (Callinan and Room, 2023; Morris et al., 2023a). Specifically, continuum beliefs have been associated with greater problem recognition amongst larger non-help seeking AUD groups (Morris et al., 2020) who are particularly deterred by the stigma-laden alcoholic label (Morris et al., 2021) and its implications for the self and recovery (Dar-Nimrod et al., 2013; Morris, 2022; Piras et al., 2016; Wallhed Finn et al., 2014).

In this regard, a continuum model has clear implications for *who* is perceived as having ‘lived experience’ of alcohol problems, and indeed *what* recovery is. Typically, lived experience of AUD is recounted by those with the most severe forms of AUD via recovery-orientated discourse (Morris et al., 2022). This skew towards higher severity lived experience accounts likely represents the aforementioned historical and social (i.e., stigma) drivers of ‘alcoholism’ models which have evolved through a reifying process of the *availability heuristic* (Tversky and Kahneman, 1973). That is, as various iterations of disease model discourse spread, an alcoholism-orientated paradigm became received wisdom, in turn leaving an *explanatory vacuum* for more nuanced understandings of alcohol problems and their resolution (Morris et al., 2020; Oettingen et al., 2006). For instance, readers will likely be able to recall instances of people with lived experience sharing powerful addiction recovery stories through a disease model narrative, whether at conferences, through media, or in fiction. However, we suggest readers are less likely to have encountered – and if so *recall* – accounts of natural or non-abstinent recovery relayed as lived experience of AUD. This comes despite decades of evidence demonstrating the importance of both natural and non-abstinent recovery, even within the ‘clinical world’ of more severe AUD populations (Henssler et al., 2020; Witkiewitz et al., 2020; Witkiewitz and Tucker, 2020). The dominant alcoholism master narrative has thus arisen out of a quasi-scientific but cognitively and socially attractive public motivation to distil and essentialize AUD to the ‘alcoholic other’, in turn setting a high threshold for what counts as AUD in the public mindset. Further, this paradigm has been bolstered by alcohol industry interests who benefit from framing AUD as a problem of a distinct biological minority, thus undermining the value of population level responses that would be harmful to their commercial interests (Bhattacharya et al., 2018; McCambridge et al., 2021; Room, 2001). ‘Alcoholism’ and its embedded heuristics of disease, genes, severity and abstention-as-recovery have thus come to form a *collateral reality* (Law, 2013) in which competing frames^4^ of alcohol problems have largely failed to resonate. As such, and consistent with a Kuhnian paradigm in which competing narratives are subverted (Kuhn, 1970), experiences of non-abstinent and natural recovery have been ignored, underplayed, or even actively discredited. This is most strongly exemplified by the smears on controlled drinking research beginning in the 1960 s (Roizen, 1987; Sobell and Sobell, 1995) and still commonly reflected in lay discourse today (Atkinson et al., 2023; Coulson, 2014).

Compared to a biomedical model, a continuum model of AUD also aligns with the distribution of consumption model, whereby the smoothness of the distribution across populations implies there are no cut-offs between ‘problem’ and ‘non-problem’ drinkers (Johnstone and Rossow, 2009). Indeed, it has been suggested that *heavy use over time* might be sufficient to capture alcohol-related problems and would do so in a less stigmatizing way (Rehm et al., 2013). Viewing alcohol use and problems on a continuum also better aligns with universal prevention strategies which avoids the problems associated with categorizing AUD (e.g., stigma) since their aim is to reduce alcohol consumption across the entire population.

Although an alcoholism-orientated master narrative within public discourse^5^ may assist those with *more severe* AUD to realize abstinence-based recovery, its dominance has been argued to obscure other depictions of AUD that are more conducive to aiding natural recovery and non-abstinent goals (Morris et al., 2022). The absence of a continuum model of understanding may also go some way to explaining the failure of efforts to implement routine brief interventions as an important public health strategy (O’Donnell et al., 2019). For instance, healthcare practitioners also overlook AUD levels below perceived ‘alcoholism’ thresholds and are apprehensive about alcohol discussions owing to stigma implications (Aira et al., 2003; Houghton and Taylor, 2021; Khadjesari et al., 2015). Nonetheless, some evidence of lay efforts to adopt continuum understandings exists, for instance via the terminology of ‘grey area drinking’ (Atkinson et al., 2023), but this appears limited in wider public discourse (Morris and Melia, 2019). As highlighted by a robust but largely failed effort to advance a continuum model by the Institute of Medicine (IoM) in 1990 (Institute of Medicine, 1990), alcohol use and problems are unequivocally heterogeneous, exist in multiple degrees of severity, and in turn reflect an extremely complex range of processes in its development and resolution. Unfortunately, the IoM report came at a time when genetic and neuroscientific approaches were on the ascension (Davies, 2018; Koob and Weiss, 1992), dominating addiction funding and research, and further embedding ideas of AUD as a biomedical and individually located problem (Midanik, 2004; Morris, 2022; Room, 2021).

More than 30 years after the IoM report, the scope of public narratives about AUD – the received wisdom that drinkers inherit – still fall considerably short of the true complexity and heterogeneity of AUD and recovery. As one illustrative anecdote, the first author has personal experience of alcohol problems which would have qualified as harmful drinking/alcohol dependence (NICE/ICD-11) and moderate AUD (potentially severe given the subjectivity of symptom interpretation) according to DSM-5. In short, the first author’s experience of’recovery’ occurred spontaneously with the onset of a specific alcohol-related health consequence^6^, leading to 8 years of unplanned abstinence and, to date, 12 years of ‘non-problematic’ but regular alcohol use.^7^. During this process they experienced comments from friends and family which were judged not of ill-intent, but included, “I don’t think you were a *real* alcoholic”, or when discussing the possibility of drinking again, “You’re going to *deliberately* relapse?! That’s a slippery slope!”.

We argue this singular example of lived experience highlights the need for a wider more continuum aligned understanding of alcohol use and problems to counter prevailing but problematic stereotypes about AUD. Developing a more inclusive and diverse master narrative about alcohol use and problems requires increasing the availability heuristics of natural and non-abstinent recovery, as well as emphasizing environmental and cultural factors as key drivers of AUD. The recent rise of positive sobriety movements are a welcome development, likely reflecting a consumer driven emergence of alternatives to AA or traditional recovery narratives (Sanger et al., 2019). However, these remain abstinence focused and in turn can only go so far in broadening understanding of the complexity of AUD and its resolution (Morris et al., 2022).^8^

One crucial specific aspect of public perception that may be improved by promoting continuum beliefs relates to perceptions of *controllability* for those perceived to have AUD. Perceived controllability is a key factor in behavioral theories^9^ with evidence to support its role in AUD behaviors (Cooke et al., 2016; Spada and Wells, 2010). Whilst difficulty controlling alcohol use is of course a valid and important component of scientific and clinical efforts to identify and measure addiction, lay perceptions of AUD controllability are in stark contrast to how contemporary choice theories of addiction understand self-regulation processes (Heather and Segal, 2016; Hogarth, 2020). Notably, in public terms, an inability to control alcohol use is deeply embedded within biomedical heuristics of AUD as pathological, permanent, severe, chronic, progressive, and with an abstinence only solution. In short, the alcoholism paradigm fundamentally invokes prognostic pessimism. Further, “being in control” of one’s drinking is a defining rationale behind low AUD problem recognition and othering (Melia et al., 2021; Morris et al., 2022), with control also implicated in moralizing neo-liberal discourses about alcohol use (Atkinson et al., 2023). In contrast, we know AUD to be heavily socio-cultural, existing on continuum(s) of severity, subject to multiple important psychological and environmental factors, and most commonly resolved via natural and non-abstinent recovery (Witkiewitz and Tucker, 2021).

We propose that challenging public perceptions about the perceived uncontrollability of AUD reflects one specific and stigmatized aspect that should be directly addressed to increase prognostic optimism and the reality of AUD as a malleable, heavily context dependent and psychological ‘problem of living’. Indeed some evidence suggests increasing the acceptability of non-abstinent goals may be a central component of the beneficial effects of continuum beliefs for increasing problem recognition in AUD (Leonhard et al., 2022). Further, self-efficacy has consistently been found to be central to behavior change including in AUD (Adamson et al., 2009; Cooke et al., 2016; Witkiewitz et al., 2022) and as a function of self-stigma (Schomerus, et al., 2011). Thus, a paradigm shift should be sought which, for individual-level framings, promotes a continuum model to foster nuanced and growth mindset beliefs about AUD and its resolution, whilst emphasizing the power of environmental and commercial drivers in shaping population level consumption and harms.

## 6. Recommendations for advancing a public health-first approach to reducing alcohol harms

There is no doubt that shifting the public’s ‘alcoholism’ master narrative will take time and can only go so far in reducing alcohol-related harms and AUD prevalence. Nonetheless, we argue that doing so goes hand in hand with key evidence based public health policies. Pricing, availability and marketing are the most important levers available for addressing alcohol-related harms at the population level (Burton et al., 2017; Williams et al., 2018), yet are currently heavily under-utilized owing largely to political unpopularity, in turn reflecting skeptical attitudes and misunderstanding towards public health approaches and AUD (McCambridge et al., 2014; Rutter and Glonti, 2016). Indeed, global alcohol policy has been subject to significant alcohol industry lobbying, framing strategies and legal tactics that have placed emphasis on the individual as either ‘personally responsible’ for their behavior, or as a distinct sub-set of ‘problem’ drinkers (Hessari et al., 2019; Maani Hessari and Petticrew, 2018; McCambridge et al., 2020, 2021; Morris et al., 2023; Room, 2001).

As such, alcohol policy has been proposed as one of several key issues within a commercial determinants of health agenda in which the strategies and actions of global industries have come with a heavy cost to global health outcomes (Kickbusch et al., 2016; Lee, 2023). Re-focusing towards a continuum-aligned public master narrative does not mean absolving people’s responsibility for their actions, but that any focus on responsibility should be separated from stigmatizing attitudes of blame embedded within current alcoholism tropes and narratives (Morris and Schomerus, 2023; Pickard, 2017). Further, responsibility also must be first placed on the role of society (including through the regulation of corporate interests) to protect and support individuals, for instance, as proposed by a *dynamic model of responsibility* (Schomerus et al., 2022; Schomerus and Corrigan, 2022).^10^ The need to act appears more pressing as alcohol-associated liver disease deaths and consumption continue to rise in the wake of the COVID-19 pandemic across many countries, with particular concern about the effects on those with pre-existing AUD (Angus et al., 2022; Foster et al., 2021; Gao et al., 2023; Irizar et al., 2021; Kilian et al., 2023).

We therefore call for stakeholders – including those in policy, research, advocacy, and the media – to proactively attend to how AUD is understood and represented. First and foremost, recognizing the value of person-first language and avoiding ‘alcoholism’ framings (while respecting people’s right to self-label) is not word-policing, but supports prognostic optimism and avoids the priming of harmful implicit negative stereotypes (Ashford et al., 2018; Morris and Schomerus, 2023). Many journals and addiction organizations have taken steps to change stigmatizing terminology, but it remains prevalent in many domains of addiction discourse outside of self-labelling contexts, including within addiction research (Hartwell et al., 2022). Attending to language therefore represents an important early-stage strategy which shapes and influences thought processes and decision making from policy makers down to individuals. This supports calls for evidence based stigma-reduction strategies of person-first language, humanizing narratives and an emphasis on societal over individual drivers of addiction (McGinty and Barry, 2020).

In terms of alcohol and AUD research, we recommend that significant attention is paid to the balance of funding and emphasis towards different disciplines with consideration of what will yield the greatest reductions in alcohol-related harms and improvements in overall wellbeing. As we have set out, funding and research has been heavily weighted towards biomedical etiological factors which have led to little advancement in AUD treatment and prevention over many decades. This is not to argue for an end to biomedical research, but for acknowledgement of this position and an intention to ‘rebalance the bio’ in a so-called biopsychosocial model (Morris et al., 2023). Specifically, we propose some key under-addressed areas of research with strong potential to reduce the harms associated with AUD and improve policy and treatment that include: evidence-based stigma reduction strategies, framing (e.g., continuum beliefs) implications for effective policy support and AUD behavior change, and actions to assist natural and non-abstinent recovery agendas.

We also advocate for the development of AUD concepts/models that go beyond disease-based conceptualizations to better reflect the heterogeneity of AUD (Boness et al., 2021; Litten et al., 2015). One example is the Etiologic Theory-Based Ontogenetic Hierarchical Framework (ETOH) of AUD, a systematic integration of the evidence related to the processes that cause and maintain AUD (Boness et al., 2021). Whilst this framework acknowledges processes such as loss of control, it also incorporates other empirically relevant processes such as coping and drinking motives. Although the ETOH is lacking in explicit consideration of social and environmental contributors to AUD, it serves as a starting point for going beyond disease-based conceptualizations of AUD in understanding etiology as well as in research, diagnosis/classification, and treatment efforts. Frameworks that more explicitly account for the inherent complexity of AUD can also support other recommendations made here, such as supporting continuum-aligned models to increase problem recognition and understanding of natural recovery. Further, these may assist in minimizing sociopolitical influences on AUD conceptualizations and diagnostic criteria which can undermine prevention and treatment efforts (Boness et al., 2022).

## 7. Conclusion

Significant biases and limitations exist in both professional and public understandings regarding the nature of alcohol use and problems. This in turn has important implications for prevention and recovery across structural (e.g., in funding, research and policy), societal (e.g., public attitudes including stigma), and individual levels (e.g., for problem recognition and ‘recovery’). Differences across contemporary professional conceptualizations of AUD reflect the inherent complexities and challenges of developing accurate and useful AUD ontologies, limitations to current scientific understanding, and historical and sociocultural influences. These biases and limitations reflect a historically embedded disease-orientated ‘alcoholism’ paradigm which has been aided by a range of coalescing factors including the worldwide recognition of AA, a biomedical focus on understanding AUD, cognitive biases including reductionism and essentialism, and various societal and commercial motives to construct AUD problems as confined to the *biogenetic other*. To address these concerns, we advocate for a renewed public-health first approach to alcohol-related harm that focuses on commercial determinants and upstream policies which aim to reduce drinking across the entire distribution of drinkers, continuum models of use and harms, stigma reduction approaches, and further research to support refinement of AUD concepts to facilitate improved prevention and treatment approaches.

## Footnotes

1 While some studies have identified some positive stigma effects of disease or biomedical attributions towards AUD (e.g., Schomerus et al., 2013, 2014b), other studies have indicated null or negative effects of disease or biomedical attributions on AUD stigma (e.g., Piras et al., 2016; Rundle et al., 2021; Wiens and Walker, 2015), particularly when compared to psychosocial attributions.

2 Whilst highlighting here how AA texts promote an idea of fundamental difference between ‘alcoholics’ and others, we acknowledge how this may be both beneficial to members, and the significant variation in how AA members individually interpret and apply AA concepts, albeit a distinct ‘alcoholic identity’ appears a ‘non-negotiable’ aspect (Glassman, Moensted, et al., 2022).

3 Whilst the precise nature of a continuum model for alcohol use and problems warrants further exploration, it is mainly based on the absence of group categories or fixed thresholds (whilst acknowledging the pragmatic case for categorizations such as in clinical contexts). See Morris et al. (2023) for further discussion.

4 According to Critical Frame Analysis (Verloo, 2007), frames may function to determine or reinforce dominant paradigms such that discourse or communication not aligned with dominant cultural frames can be subverted or overlooked owing to not being understood or accepted (Entman, 1993; Gamson, 1992). For instance, Luntz et al. (2023) state in the context of medicalization of health, “long-standing ideas of personal responsibility in public discourse become accessible reservoirs of counterarguments when alternative narratives are presented” (p. 73).

5 Little quantitative data exists to support this, although one study amongst a German sample found 27% of respondents agreed with a continuum model of AUD, whilst 40% disagreed and 30% were undecided (Schomerus et al., 2013).

6 Specifically, alcohol-related gastritis. It is worth noting the first author also experienced various social and functioning costs which did not induce any contemplative processes, likely due to the normalization of heavy drinking in their social networks and cultural acceptability of ‘binge drinking’ around the millennium.

7 Readers may be curious to know how the first author defines his use as regular but non-problematic. Over 12 years the broad pattern of use averages 3 weekly drinking occasions, averaging a total of around 10–20 units per week. The lead author does not consider this risk free, as any level of consumption carries some risks, but has not experienced any indicators of dependence or previously experienced harmful consequences (though has experienced some negative effects such as interrupted sleep).

8 This is not to suggest abstinence goals are not best - or indeed necessary - for some AUD groups, including, for instance, anyone with liver disease.

9 Notably in the Common Sense Model of Illness Representations (Hagger and Orbell, 2003) and Theory of Planned Behavior (Ajzen, 1991)

10 Specifically, a dynamic model of responsibility suggests that with increased severity of AUD, greater emphasis should be placed on the role of society, not the individual, in part to attenuate the heavy individual stigma consequences of severe AUD
